# Ultrasound‐Assisted Precise *In Situ* Decompression for Cubital Tunnel Syndrome

**DOI:** 10.1111/os.12922

**Published:** 2021-03-21

**Authors:** Jin‐mei Gao, Yu Yuan, Ke‐tong Gong, Xin‐long Ma, Xin Chen

**Affiliations:** ^1^ Department of Ultrasound Tianjin Hospital Tianjin China; ^2^ Department of Hand microsurgery Tianjin Hospital Tianjin China; ^3^ Department of Orthopaedic Surgery Tianjin Hospital Tianjin China; ^4^ EMG Room Tianjin Hospital Tianjin China

**Keywords:** Cubital tunnel syndrome, Treatment effect, Ulnar nerve, Ultrasonography

## Abstract

**Objective:**

To explore the effect of locating the ulnar nerve compression sites and guiding the small incision so as to decompress the ulnar nerve *in situ* on the elbow by high‐frequency ultrasound before operation.

**Methods:**

A retrospective analysis was conducted on 56 patients who underwent ultrasound‐assisted *in situ* decompression for cubital tunnel syndrome from May 2018 to August 2019. The patients' average age was 51.13 ± 7.35 years, mean duration of symptoms was 6.51 ± 1.96 months, and mean postoperative follow‐up was 6.07 ± 0.82 months. Nine patients had Dellon's stage mild, 39 had stage moderate, and eight had stage severe. Ultrasound and electromyography were completed in all patients before operation. The presence of ulnar nerve compressive lesion, the specific location, and the reason and extent of compression were determined by ultrasound. A small incision *in situ* surgery was given to decompress the ulnar nerve according to the pre‐defined compressive sites.

**Results:**

All patients underwent *in situ* decompression. The compression sites around the elbow were as follows: two in the arcade of Struthers, one in the medial intermuscular septum, four in the anconeus epitrochlearis muscle, five beside the cyst of the proximal flexor carpi ulnaris (FCU), and the remaining 44 cases were all from the compression between Osborne's ligament to the two heads of the FCU. The compression localizations diagnosed by ultrasound were confirmed by operations. Preoperative ultrasound confirmed no ulnar nerve subluxation in all cases. The postoperative outcomes were satisfactory. There was no recurrence or aggravation of symptoms in this group of patients according to the modified Bishop scoring system; results showed that 43 cases were excellent, 10 were good, and three were fair.

**Conclusions:**

High‐frequency ultrasound can accurately and comprehensively evaluate the ulnar nerve compression and the surrounding tissues, thus providing significant guidance for the precise minimally invasive treatment of ulnar nerve compression.

## Graphical abstract

The ulnar nerve became thinner at the site of Osborne's ligament in the longitudinal section. The accurate site of ulnar nerve compression could be located by high‐frequency ultrasound, that is the important foundation for the precise *in situ* ulnar nerve decompression.

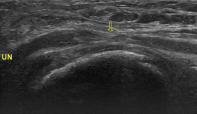

## Introduction

Cubital tunnel syndrome is symptomatic ulnar nerve dysfunction at the level of the elbow that results from a combination of compression, traction, and friction. Most cases are idiopathic, and symptoms consist of a combination of weakness, pain, and sensory disturbances ranging from paresthesia and dysesthesias to numbness or complete anesthesia. Also, the hand may start developing a claw deformity due to intrinsic muscle weakness and the unopposed function of the flexor digitorum profundus. Untreated chronic cubital tunnel syndrome can lead to permanent loss of sensibility and secondary joint contractures. Ulnar nerve entrapment is the second most common compression neuropathy in the upper extremity after carpal tunnel syndrome^1^. The cubital tunnel syndrome mainly forms because of long‐term and repeated elbow flexion that moves the ulnar nerve, thus increasing the internal tension of the cubital canal, which in turn affects the microcirculation in the nerve and ischemia and hypoxia cause nerve damage^2^. The early lesions of cubital tunnel syndrome can be treated with splinting at night time, thus keeping the elbows straight, medication, partial closure, and electro‐acupuncture^3^. Patients with severe signs and symptoms such as atrophy of interossei and weakness of handgrip strength might not improve with conservative management. Also, patients for whom conservative treatment has failed would require surgical intervention to improve their symptoms^4^.

Although nonsurgical management is recommended in the majority of these cases, 42% of patients eventually require surgical release^5^. Over recent years, the rate of surgical management of cubital tunnel syndrome has increased in the United States^1^. The same trend was observed in England, where the rate increased from 31% to 67%^6^. Early surgical treatment could maximize the protection of hand function and reduce muscle atrophy. The key to treatment is to clarify the cause of compression and remove the factors causing compression. Currently, the widely used surgical method is ulnar nerve transposition^4^, ^7^. Anterior transposition of the ulnar nerve relieves both nerve compression and strain by moving the ulnar nerve anterior to the ulnohumeral axis. This procedure can completely remove all potential compressive sites around the elbow joint at one time; however, the extent of damage is large. The scar caused by surgery is long, and there is a risk of new compression. Another surgical treatment is *in situ* decompression, which in the past used to be done fully open. In contrast, recently, smaller incisions are being used more frequently to minimize recovery time and improve outcomes^8^. The theoretical benefits of small incision cubital tunnel *in situ* decompression include the reduction in nerve handling, surrounding tissue dissection/trauma, expedited recovery time for patients, reduced vascular complications, and decreased scar discomfort. Such decompression of the ulnar nerve *in situ* can only solve lesions at 1–2 compressive sites due to the small incision. Therefore, the compression sites must be determined before surgery, and other potential compression sites must be accurately evaluated so as to correctly select the appropriate surgical indications for precise treatment of ulnar nerve compression. According to the clinical examinations, the accuracy of determining the compression sites is not high. Some studies have achieved good results using electromyography to locate the compression sites^9^. Still, the electromyography can only locate a general range, and the cause for the compression cannot be determined. Moreover, the patients do not tolerate the invasive examination well.

Technological advances in ultrasonography have allowed for direct visualization of the involved nerve with an assessment of the exact site and extent, which yielded unmatched information about anatomical details of the nerve. The purposes of this study were as follows: (i) to evaluate the accuracy of locating the ulnar nerve compression sites by high‐frequency ultrasound; (ii) to explore the feasibility of the ulnar nerve decompression guided by preoperative ultrasound; and (iii) to evaluate the therapeutic effect of ultrasound‐assisted ulnar nerve precise *in situ* decompression by modified Bishop's scoring system. We found that ultrasound is a good auxiliary tool, which can locate the compression sites of the ulnar nerve accurately and detect the subluxation of the ulnar nerve before operation, which provides minimally invasive treatment for cubital tunnel syndrome.

## Materials and Methods

### Population

This retrospective analysis included a total of 56 patients with cubital tunnel syndrome who underwent *in situ* decompression surgery in our hospital between May 2018 and August 2019. Inclusion criteria: (i) age >18 years; (ii) clinically evident ulnar nerve palsy: sensory disturbance in the ulnar half of the fourth and fifth fingers as well at the palmar or dorsal aspect of the hand and/or weakness of hand muscles innervated by the ulnar nerve; (iii) EMG: motor conduction velocity (MCV) across the elbow <50 m/s; (iv) ultrasound: ulnar nerve was compressed around the cubital tunnel and the maximum cross‐sectional area ≥0.10 cm^2^; and (v) signs and symptoms present for>3 months.

Patients were excluded if: (i) they had tumor compression in the ulnar nerve or tumor of the nerve itself; (ii) obvious compression caused by arthritis such as rheumatoid arthritis and tuberculosis of elbow joint; (iii) obvious osseous hyperplasia and deformity of elbow joint; (iv) subluxation of the ulnar nerve in the cubital tunnel by dynamic ultrasound observation of elbow flexion; and (v) previous surgery on the symptomatic elbow.

This retrospective study was approved by our institutional review board. All patients signed informed consent for the use of their medical data. After the ultrasound examination, patients received local small incisions. A total of 56 patients, including 44 males and 12 females, aged 26–67 years (average, 51.13 ± 7.35 years) were selected and followed up successfully. All of them were diagnosed with unilateral cubital tunnel syndrome, with 23 cases on the left side and 33 cases on the right side. In this study, the patients' preoperative clinical manifestations were determined with Dellon's staging system^10^: there were nine mild cases, 39 moderate cases, and eight severe cases (Table 1). The average duration of symptoms was 6.51 ± 1.96 months (range, 3–20 months). The patients were instructed to take examination and electromyography 6 months after operation. Average follow‐up after surgery was 6.07 ± 0.82 months (range, 5–7 months).

**TABLE 1:** Dellon's classification of cubital tunnel syndrome in patients

	Mild (I)	Moderate (II)	Severe (III)
Sensory	Intermittent paresthesia	Intermittent paresthesia	Permanent paresthesia
Motor	Subjective weakness	Measurable weakness	Palsy
Number of patients	9 (16.1%)	39 (69.6%)	8(14.3%)

### Ultrasonic Measuring

The ultrasound examinations were performed by two radiologists with experience in musculoskeletal ultrasound using the GE LOGIC E9 ultrasound device (GE Healthcare, Milwaukee, WI, USA) with ML6–15 linear transducer (frequency: 6.0–15.0 MHz) (GE Healthcare, Milwaukee, WI, USA). The ulnar nerve was examined with the patient in the supine position, where the upper limb of the tested side was naturally placed on one side of the body and the palm was slightly rotated forward. The axial scanning and longitudinal scanning method were used to scan one side of the ulnar nerve continuously. The range of examination included the whole course of ulnar nerve, the proximal end started from axillary region, and the distal end should reach the level of the Guyon's canal. The morphology, echo, and cross‐sectional area of the ulnar nerve were observed. Cross‐sectional area measurements were made at the point of maximal enlargement near cubital tunnel, and the measurements were performed by using the trace function of the ultrasound device. Furthermore the compressing factors were evaluated. Finally, it was very important to evaluate whether the ulnar nerve subluxation at the maximum flexion position of the elbow.

### Ultrasound Diagnosis of Cubital Tunnel Syndrome

In the longitudinal section of the normal ulnar nerve, there were hypoechoic bands with the same thickness, and parallel linear hyper‐echogenicity was seen in the interior. In the transverse section, the hypoechoic was oval or round, surrounded by high echo envelope structure, and the internal echo was multiple round hypoechoic, surrounded by slightly hyperechoic, and showing a honeycomb appearance. When the ulnar nerve was compressed, the space‐occupying structure beside the nerve could be seen. The compressed point of the ulnar nerve becomes thinner, and the proximal ulnar nerve becomes enlarged and edematous with an increase in echogenicity with loss of fascicular pattern. The maximum cross‐sectional area was enlarged^11^. Ultrasound examination was used to determine the cause of compression and to record which ligament structure was bound to the surface of nerve compression. If the ulnar nerve slips to the front of the medial condyle of humerus in the elbow flexion position, there was ulnar nerve subluxation.

### Electromyography

The third examiner (clinical neurophysiologist >15 years) performed ulnar electromyography examination across the elbow pre‐ and post‐operation using a standard EMG system (Nicolet Synergy, Natus Medical Incorporated, San Carlos, USA). Electromyography of the FCU, abductor digiti minimi, and first dorsal interosseous muscle were performed in all patients. The motor conduction velocity (MCV) of the ulnar nerve in three segments (axilla‐above the elbow, below the elbow‐wrist, and above the elbow‐below the elbow) was evaluated in all patients. Segmental decrease in MCV usually indicates the range and extent of nerve entrapment.

### Surgical Procedures

In our study, wide‐awake local anesthesia was used in all cases. According to the compression site indicated by ultrasound before the operation, the local *in situ* decompress operation was performed on the entrapment factors. The ulnar nerve was decompressed without anterior transposition. The incision length was about 3–4 cm. Osborne's ligament was cut off in most of the elbow segments. According to the situation, the entrance of FCU was released, and the synovial cysts were cleared. If the anconeus epitrochlearis muscle was involved, the accessory muscle was removed, and the frenulum of the ulnar nerve was reserved to prevent subluxation.

### Outcome Measures

Postoperative clinical outcome of symptomatology and degree of improvement was assessed in all patients based on a modified Bishop scoring system^12^: excellent score was above 8 points out of the 12 points scoring system, good was between 6 and 7, fair was between 4 and 5, and poor was below 3 (Table 2).

**TABLE 2:** Modified Bishop's scoring system

Items	Score
Residual symptoms	
None	3
Little/Intermitted	2
Moderate	1
Severe	0
Subjective improvement	
Better	2
Unchanged	1
Worse	0
Ability to work	
Working in old job	2
Changed job due to complaints	1
Incapable of working	0
Muscle strength	
Better	1
Unchanged	0
Sensory disturbance	
Better	1
Unchanged	0
Evaluation	
Excellent	8–9
Good	6–7
Fair	4–5
Poor	≤3

### Statistical Analysis

EpiData 3.1 software (The EpiData Association, Odense, Denmark) was used for data input, and SPSS 17.0 software (SPSS Inc., Chicago, IL, USA) was used for statistical analysis. The continuous data were expressed by mean ± standard deviation (SD). The mean values of the same group before and after operation were compared by self‐paired *t*‐test. A *P*‐value of ≤0.05 was considered statistically significant.

## Results

### Results of Preoperative Examination

#### Ultrasound

**Fig. 1:**
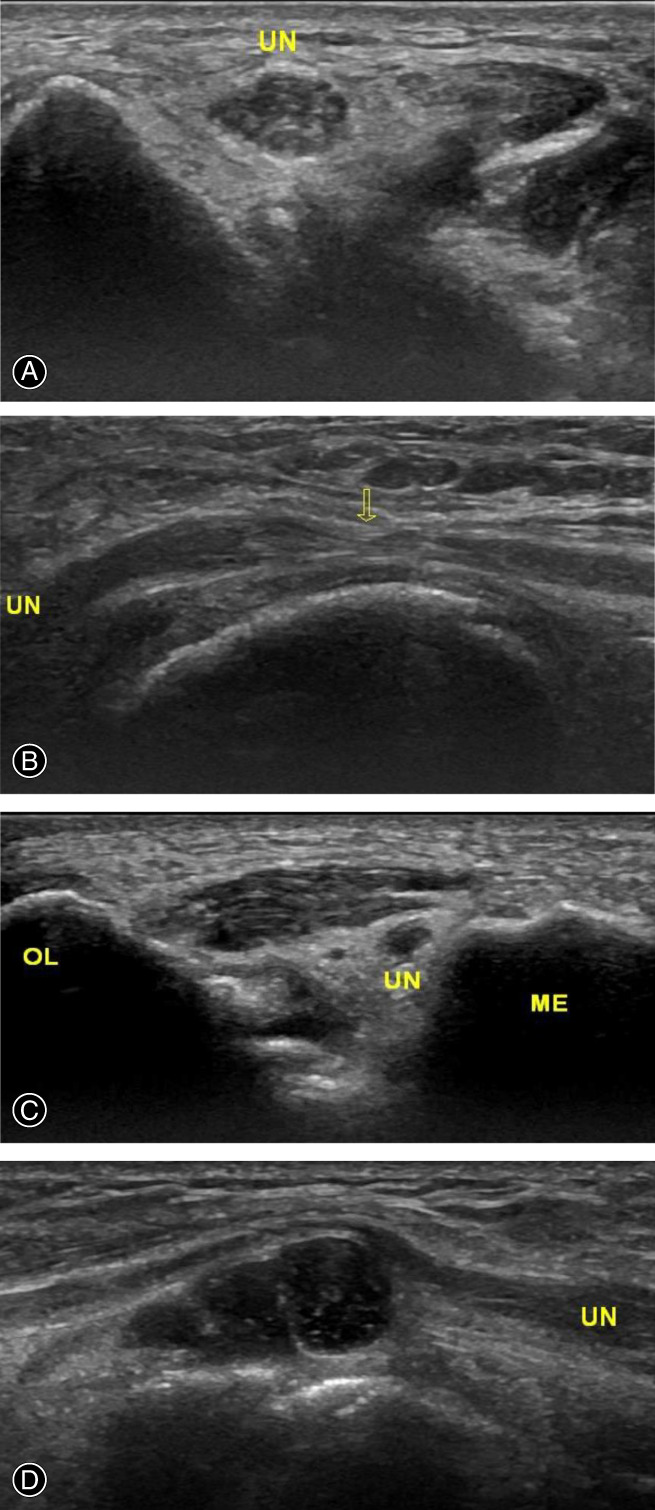
Ultrasound measure results before the operation. (A) The ulnar nerve thickened at the proximal end of the cubital canal in the transverse section. (B) The ulnar nerve became thinner and thicker at Osborne's ligament in the longitudinal section. (C) Compression of the ulnar nerve by anomalous anconeus epitrochlearis muscle in cross‐section. (D) The ulnar nerve was compressed by a deep square cyst in the longitudinal section. UN, Ulnar Nerve; OL, Olecranon; ME, Medial Epicondyle of Humerus.

The ulnar nerve changed in thickness from uniform to local thinning at the compression site when the ulnar nerve was compressed, and the nerve proximal to compression swelled segmentally with the hypoechoic. The transverse section showed that the structure of the internal nerve bundle was unclear, and the cross‐sectional area was increased. An ultrasound examination was completed within 1 week before the operation. The proximal ulnar nerve was thickened to varying degrees. The maximum cross‐sectional area was 0.11–0.26 cm^2^, with an average of 0.16 ± 0.05 cm^2^. Ultrasound could diagnose the presence of compression, as well as accurately locate and identify the reason for compression (Fig. 1A–D). The entrapment sites around the cubital tunnel included one case at the medial muscular septum, two cases at arcade of Struthers, four cases at anconeus epitrochlearis muscle, five cases beside the cyst of the proximal FCU, and 44 cases localized in a tiny region from Osborne's ligament to two heads of FCU.

#### Electromyography (EMG)

Electromyography (EMG) was performed at the same time as preoperative ultrasound (interval < 1 week). EMG showed that the MCV of the ulnar nerve in the elbow of the affected side was slowed down (21.9–48.5 M/s), with an average of 43.31 ± 7.64 M/s. MNCV across the elbow <50 m/s indicated ulnar neuropathy at the elbow, according to the AAEM guidelines^13^.

### Intraoperative Findings

All operations were performed with wide‐awake local anesthesia. The incision length was 3–4 cm. In 56 patients, the coincidence rate of intraoperative entrapment with preoperative ultrasound was 100%. The localized compression site of the ulnar nerve indicated by preoperative ultrasound was decompressed *in situ*, and other segments without entrapment indication by ultrasound were not explored during the operation. The frenulum of the ulnar nerve should be preserved to protect the blood supply of the superior ulnar collateral artery. The compressive fascial bands should be decompressed without neurolysis to avoid destabilizing the ulnar nerve.

### Postoperative Follow‐up

#### Complications

All patients were followed up for 5 to 7 months (6.07 ± 0.82 months), and the effect of a small incision *in situ* decompression was satisfactory. Neither severe complications nor recurrences were observed during the follow‐up period. The postoperative complications contain two cases of hematoma, one case of infection, and one case of medial antebrachial cutaneous nerve injury. Since the complications were not serious, no specific treatment was given. The symptoms of complications disappeared at the time of 6‐months follow‐up. No obvious change in ulnar nerve entrapment symptoms was reported in only three patients; however, their muscle atrophy did not progress. Also, the numbness around the little finger was significantly relieved or completely disappeared in 53 patients.

#### Bishop Score

According to the modified Bishop scoring system, 43 cases were evaluated as excellent, 10 cases were good, and three cases were fair (Table 3). A total of 94.6% (53/56) cases achieved an “excellent” or “good” Bishop score and none of the 56 patients needed reoperation.

**TABLE 3:** Outcomes of Bishop's scores in terms of Dellon's classes

	Excellent	Good	Fair	Poor	Preoperative MCV (m/s)	Postoperative MCV (m/s)	*T*‐value	*P‐*value
Dellon I (n = 9)	9	0	0	0	45.81 ± 4.28	51.19 ± 4.76	5.26	0.00
Dellon II (n = 39)	30	8	1	0	43.46 ± 5.27	49.93 ± 5.02	6.63	0.00
Dellon III (n = 8)	4	2	2	0	31.63 ± 5.72	40.71 ± 5.46	7.38	0.00
Total (n = 56)	43	10	3	0	43.31 ± 7.64	49.65 ± 7.36	6.92	0.00

#### Motor Conduction Velocity (MCV)

Postoperative electromyography follow‐up results indicated that the average motor conduction velocity (MCV) of the ulnar nerve in the elbow was 49.65 ± 7.36 m/s, which was statistically increased when compared with before operation (*P* < 0.05). There was no ulnar nerve subluxation after an operation.

## Discussions

Cubital tunnel syndrome is caused by compression of the ulnar nerve in the cubital canal that occurs due to various reasons. The common parts of the ulnar nerve compression caused by the elbow are scattered in the 5 cm area above and below the elbow joint, and located on medial intermuscular septum, arcade of Struthers, medial epicondyle, the Osborne ligament, the two heads of the FCU, and deep flexor pronator aponeurosis distally^14^. The typical manifestation is “claw hand” deformity. The clinical diagnosis of cubital tunnel syndrome is relatively clear, and typical clinical symptoms and electromyography can be used to confirm the diagnosis. If conservative treatment is ineffective, surgical treatment should be considered^15^. There are many surgical treatment methods for cubital tunnel syndrome, which could be divided into the following two categories: ulnar nerve *in situ* decompression, which includes open surgery and endoscopic surgery. There are two kinds of *in situ* decompression of medial humeral epicondyle resection and non‐resection^16^, ^17^. The second type is the anterior transposition of ulnar nerve^18^. At present, an anterior transposition is widely used, and it includes submuscular, intramuscular, or subcutaneous transposition of ulnar nerve. Among these methods, subcutaneous transposition of the ulnar nerve is the most commonly used method because of its simple operation and reliable curative effect.

During the surgical treatment, it is usually necessary to free up to the medial intermuscular septum all the way down to the outlet of the FCU so as to completely relieve the compression, which is why the surgical incision is usually 15 cm. All compression points of the ulnar nerve can be loosened during the operation. Although the decompression is complete, the operation is traumatizing, and the postoperative recovery is slow. Bartels *et al*.^19^ reported that the operation time of ulnar nerve anterior transposition takes a longer time and the postoperative complications are more common. Recent studies have shown that *in situ* decompression of the ulnar nerve could also achieve better results^20^, ^21^. In a multicenter retrospective study, 375 patients were followed up for an average of 92 months after surgery to compare the effects of four surgical techniques (open or endoscopic *in situ* decompression, subcutaneous or submuscular anterior transposition). There was no significant difference in the efficacy of the four surgical methods, and the symptoms improved in more than 90% of patients. In addition, the long‐term complications and recurrence rate were very low^22^. A recent meta‐analysis^23^ included 2154 procedures. Of these, 1040 were *in situ* simple decompression, and 1114 were ulnar nerve transposition procedures. There is no statistically significant difference in clinical outcomes or rate of revision surgery between simple decompression *vs* ulnar nerve transposition.

Since the position of ulnar nerve compression is not just in one site, the small incision decompression of the cubital tunnel may result in missing the compression of other positions. Although this is not a common occurrence, it could directly affect the treatment effect. Therefore, the accurate positioning of the compression site before surgery becomes essential for successful *in situ* decompression surgery. Electromyography examination of the motor nerve conduction velocity of 5 cm above and below the cubital tunnel has a clear diagnostic value for the cubital tunnel syndrome; yet, it is somewhat traumatic. The cause of entrapment cannot be determined, and accurate positioning cannot be achieved because electromyography cannot provide the imaging information of nerves and surrounding tissues. Besides, previous studies have reported that electromyography is less sensitive in the diagnosis of cubital tunnel syndrome compared with carpal tunnel syndrom, and there are some false negatives, which is not a “gold standard” for diagnosis of cubital tunnel syndrome^24^, ^25^. According to a previous report where electrical stimulation was performed at intervals of 1 cm to measure the conduction velocity^26^, inching technique can reduce the compression range from 4 cm below the elbow to 6 cm above the elbow. Nevertheless, this method is more cumbersome in operation, and it is impossible to identify the cause of the entrapment.

Although MRI has high diagnostic sensitivity and specificity, it is expensive and time‐consuming. The equipment popularity rate is not high, the fine resolution of soft tissue is not as good as in high‐frequency ultrasound, and it cannot be used for dynamic observation. Following the development of ultrasound diagnostic equipment and the improvement of musculoskeletal ultrasound diagnosis, the use of high‐frequency ultrasound for the examination of peripheral nerve injury and nerve entrapment diseases has been gradually popularized because it provides anatomic information not available with electrodiagnostic studies, is readily available, is inexpensive, does not involve radiation exposure, and is painless. The most significant advantage of high‐frequency ultrasound examination of peripheral nerves is that it can visually display nerve morphology, nerve travel, and compression position, thus providing accurate imaging information for clinical diagnosis and surgical treatment.

After the peripheral nerve is compressed, chronic ischemia, hypoxia, and increased vascular permeability cause nerve edema. If the compression factor is not removed, a vicious cycle of ischemia, hypoxia, and edema might form, as well as the nerve fiber tissue hyperplasia and nerve Wallerian degeneration of fibers^27^. The thickening of the proximal end of the compression and the thinning of the compression point form a nerve notch on the ultrasound image of the longitudinal section. On the ultrasound image of the transverse section, the segmental shape change of the ulnar nerve from circular to elliptical could be observed. The echo of the beam is reduced, the beam‐like structure is blurred, the echo of the outer membrane and the beam membrane is enhanced, and the cross‐sectional area is increased. Different studies reported different diagnostic cut‐off values of cross‐sections. In this study, the diagnostic criteria were CSA ≥0.10 cm^2^. Volpe *et al*.^28^ reported that the diagnostic cut‐off values were more than 88% sensitive and specific for the diagnosis of cubital tunnel syndrome. Agarwal *et al*.^29^ reported that ultrasound had an excellent diagnostic effect on ulnar nerve compression, and the abnormal morphological findings were consistent with what was seen during the operation, which suggests ultrasound diagnosis of cubital tunnel syndrome has important reference value for the staging of ulnar neuropathy and treatment methods choices. In this study, all the compression sites diagnosed by preoperative ultrasound were confirmed during the operation and the localization diagnosis accuracy was 100%, which was consistent with the former reports.

Accurate localization with ultrasound was helpful to make the operation plan in advance. The small incision *in situ* decompression may achieve the effect of large‐scale decompressing of all potential compression sites. A total of 94.6% (53/56) of cases in this study achieved an “excellent” or “good” bishop score, which was slightly higher than the improved rate reported in the literature^21^, ^23^. Furthermore, there were significantly less complications by applying the ulnar nerve *in situ* decompression^21^, ^23^. The complications in this study were 7.1% (4/56), slightly lower than those reported in the literature.

Postoperative nerve subluxation is the most common cause of cubital tunnel surgery failing^30^, ^31^. The overall incidence of anterior instability in the setting of simple decompressions ranges from 2.4% to 17.0% of cases^32^. The effect in some patients, whose ulnar nerve was instable before an operation, was not good after *in situ* decompression^33^. At the same time, an anterior transposition is widely accepted as the preferred method for treating cubital tunnel syndrome where ulnar nerve subluxation is present, and *in situ* decompression should be avoided. Matzon *et al*.^34^ reported that, of 363 patients considered for *in situ* decompression treatment, 76 (21%) underwent ulnar nerve transposition owing to ulnar nerve instability; 29 patients were identified on examination before surgery, 44 were identified during surgery, and three were identified after surgery and had to undergo revision surgery. As a result, 67.8% (47/76) ulnar nerve instability was not be identified before surgery. This study used a dynamic ultrasound examination to evaluate the stability of the ulnar nerve before surgery accurately. For patients in whom ulnar nerve subluxation was detected before surgery, ulnar nerve anterior transposition was applied instead of *in situ* decompress. The frenulum of the ulnar nerve was preserved during operation by less nerve manipulation and minor dissection, therefore the possibility of postoperative nerve subluxation affecting the treatment effect was reduced.

### Limitations

The present study has some limitations. First, the group criteria for patients was limited to patients with more severe symptoms. The Dellon's classification was mainly mild to moderate, and the results were relatively good. Second, there was no randomized controlled study for clinical outcomes and complications on *in situ* decompression of ulnar nerve with and without ultrasonic assisted localization. Third, there was a short amount of follow‐up time after surgery and lack of follow‐up data that evaluated long‐term outcome. Due to the limited sample size, the obtained results might be biased. It is necessary to further increase the sample size for further comparative studies in the future.

### Conclusions

High‐frequency ultrasound can accurately and comprehensively evaluate the compression and stability of ulnar nerve, thus providing guiding significance for the precise minimally invasive treatment of ulnar nerve compression. In turn, ultrasound can be a helpful tool for surgical treatment of cubital tunnel syndrome. *In situ* decompression, assisted by ultrasound, can be an efficacious surgical method for cubital tunnel syndrome.

## Authorship Declaration

All listed authors meet the authorship criteria and are in agreement with the manuscript.
